# The NAS Perchlorate Review: Adverse Effects?

**DOI:** 10.1289/ehp.113-1310937

**Published:** 2005-11

**Authors:** Richard B. Johnston, Richard Corley, Linda Cowan, Robert D. Utiger

**Affiliations:** University of Colorado School of Medicine, Denver, Colorado; Pacific Northwest National Laboratory, Richland, Washington; University of Oklahoma Health, Sciences Center, Oklahoma City, Oklahoma; Harvard Medical School, Boston, Massachusetts, E-mail: rutiger@partners.org

Ginsberg and Rice (2005) argued that the reference dose for perchlorate of 0.0007 mg/kg per day recommended by the National Academy of Sciences (NAS) Committee to Assess the Health Implications of Perchlorate Ingestion is not adequately protective. As members of the committee, we disagree.

Ginsberg and Rice (2005) based their conclusion on three points. The first involves the designation of the point of departure as a NOEL (no observed effect level) versus a LOAEL (lowest observed adverse effect level). The committee chose as its point of departure a dose of perchlorate (0.007 mg/kg per day) that, when given for 14 days to seven normal subjects, did not cause a statistically significant decrease in the group mean thyroid iodide uptake (Greer et al. 2002). Accordingly, the committee considered it a NOEL. Ginsberg and Rice (2005) focused on the fact that only seven subjects were given that dose; they seem to say that attention should be paid only to the results in those subjects in whom there was a decrease in thyroid iodide uptake and that the results in those in whom there was no decrease or an increase should be ignored. They considered the dose to be a LOAEL because of the decrease in uptake in those few subjects. It is important to note that a statistically significant decrease of, for example, 5% or even 10% would not be biologically important and, more important, would not be sustained. For example, in another study (Braverman et al. 2004), administration of 0.04 mg/kg per day to normal subjects for 6 months had no effect on thyroid iodide uptake when measured at 3 and 6 months, and no effect on serum thyroid hormone or thyrotropin concentrations measured monthly. [Inspection of Figure 5A in Greer et al. (2002) suggests that this dose would inhibit thyroid iodide uptake by about 25% if measured at 2 weeks.]

The second issue involves database uncertainty. In clinical studies, perchlorate has been administered prospectively to 68 normal subjects for 2 weeks to 6 months. In one study (Brabant et al. 1992), a dose of 9.2 mg/kg per day for 4 weeks had no effect on thyroid function. In occupational studies, doses as high as 0.5 mg/kg per day had no effect on thyroid hormone or thyrotropin production in workers. In epidemiologic studies, there were no abnormalities in growth or thyroid function in children exposed life-long to 100–120 μg perchlorate per liter of drinking water, or in pregnant women and newborn infants similarly exposed. Given the choice of a nonadverse effect (inhibition of iodide uptake by the thyroid) as the point of departure and the multiple studies in which doses of perchlorate much higher than 0.007 mg/kg per day had no effect on any aspect of thyroid function, the committee did not apply a database uncertainty factor.

Finally, Ginsberg and Rice (2005) argued that inhibition of thyroid iodide uptake is adverse. That conclusion assumes that any acute inhibition would be sustained, so thyroid hormone production would decrease. That is not the case. There is remarkable compensation for even substantial reductions in thyroid iodide uptake—and thyroid hormone production. As noted above, subjects given 0.04 mg/kg per day for 6 months and 9.2 mg/kg per day for 4 weeks—doses that certainly would inhibit thyroid iodide uptake for a few weeks—had no decrease in serum thyroid hormone or increase in serum thyrotropin concentrations (the hallmark of even mild hypothyroidism). Short-term inhibition of thyroid iodide uptake is not an adverse effect; it has no adverse consequences because there is rapid compensation mediated by several independent processes. One of these processes is up-regulation of the thyroid sodium-iodide transport system, as a result of intrathyroidal iodide deficiency. The second, should there be even a very small decrease in thyroid hormone production, is an increase in thyrotropin secretion, resulting in overall stimulation of the thyroid gland. Analyses of the effects of any substance on thyroid function must take these compensatory processes into account, particularly the fact that the effect of any substance that inhibits thyroid function will diminish with time. Only if all of these mechanisms fail will there be hypothyroidism, the first adverse effect in the continuum of effects resulting from perchlorate ingestion. If there is no inhibition of iodide uptake to begin with, there will be no other changes in thyroid function at any time.

We believe that the committee’s recommended reference dose of 0.0007 mg/kg per day provides a wide margin of safety for all subjects of all ages.
